# The acidic protein rich in leucines Anp32b is an immunomodulator of inflammation in mice

**DOI:** 10.1038/s41598-019-41269-z

**Published:** 2019-03-19

**Authors:** Jan Chemnitz, Dorothea Pieper, Lena Stich, Udo Schumacher, Stefan Balabanov, Michael Spohn, Adam Grundhoff, Alexander Steinkasserer, Joachim Hauber, Elisabeth Zinser

**Affiliations:** 1 Heinrich Pette Institute – Leibniz Institute for Experimental Virology, Martinistrasse 52, 20251 Hamburg, Germany; 2 Department of Immune Modulation, University Hospital Erlangen, Hartmannstrasse 14, 91052 Erlangen, Germany; 3 Center for Experimental Medicine, Department of Anatomy and Experimental Morphology, University Medical Center Hamburg-Eppendorf, Martinistrasse 52, 20246 Hamburg, Germany; 4 Division of Hematology, University Hospital Zurich, Rämistrasse 100, CH-8091 Zurich, Switzerland; 5 German Center for Infection Research (DZIF), Partner site Hamburg, Hamburg, Germany

## Abstract

ANP32B belongs to a family of evolutionary conserved acidic nuclear phosphoproteins (ANP32A-H). Family members have been described as multifunctional regulatory proteins and proto-oncogenic factors affecting embryonic development, cell proliferation, apoptosis, and gene expression at various levels. Involvement of ANP32B in multiple processes of cellular life is reflected by the previous finding that systemic gene knockout (KO) of *Anp32b* leads to embryonic lethality in mice. Here, we demonstrate that a conditional KO of *Anp32b* is well tolerated in adult animals. However, after immune activation splenocytes isolated from Anp32b KO mice showed a strong commitment towards Th17 immune responses. Therefore, we further analyzed the respective animals *in vivo* using an experimental autoimmune encephalomyelitis (EAE) model. Interestingly, an exacerbated clinical score was observed in the Anp32b KO mice. This was accompanied by the finding that animal-derived T lymphocytes were in a more activated state, and RNA sequencing analyses revealed hyperactivation of several T lymphocyte-associated immune modulatory pathways, attended by significant upregulation of Tfh cell numbers that altogether might explain the observed strong autoreactive processes. Therefore, Anp32b appears to fulfill a role in regulating adequate adaptive immune responses and, hence, may be involved in dysregulation of pathways leading to autoimmune disorders and/or immune deficiencies.

## Introduction

ANP32 proteins belong to a relatively new protein family of evolutionary conserved acidic nuclear phosphoproteins (ANP32A-H). Members of this family are characterized by an amino-terminal domain, harboring several leucine-rich repeats (LRR) and an acidic carboxyterminus. The LRR structures - generating hydrophobic regions - supply *bona fide* surfaces for protein-protein interactions that may participate in intracellular signal transduction^1^. *In silico* studies identified seven human family members located on chromosomes 1, 4, 9, 12 and 15^2,3^. ANP32 proteins are not expressed in archaea or eubacteria, but are expressed in all eukaryotes. Several studies showed a high conservation of these proteins, not only between the individual family members, but also between the various eukaryotic species^4–6^. These studies imply that ANP32 proteins originate from one single ancestor gene.

Interestingly, ANP32A, B and E proteins harbor classical basic nuclear localization signals (NLS) for importin-dependent nuclear import and one or two nuclear export signals (NES) for CRM1-dependent nuclear exit^7–10^. These ANP32 family members are expressed at varying levels in several tissues and cell types. For example, in murine and human samples strong expression was detected in brain, lung, heart, kidney, muscles, intestine, stomach, liver, pancreas, leucocytes, and prostate tissue, as well as in highly proliferative organs such as the spleen, thymus and placenta^1,11–14^.

ANP32E was originally described in *Xenopus laevis* oocytes, and was subsequently also identified in murine tissues^11,14,15^. The localization and expression pattern of this protein in the cerebellum suggested a function during neuronal differentiation. Furthermore, its inhibitory potential for protein phosphatase PP2A suggested a possible overlapping function with family member ANP32A^14,16,17^. ANP32A was established as a tumor suppressor, able to activate the Apaf-1 apoptosome, and thereby inducing caspase-9 dependent apoptosis^18,19^. Furthermore, as a component of the SET complex, ANP32A can also induce apoptotic events in a caspase-independent manner^20^. In addition, as a component of the inhibitor of histone acetyl transferase (INHAT) complex, ANP32A has been reported to influence stress-induced epigenetic regulation of gene expression^21–23^. Its capacity to inhibit protein phosphatase PP2A adds another functional aspect to the various activities of ANP32A, and enables this factor to negatively influence cell cycle progression and proliferation^24–29^.

Although ANP32A is now well established as a tumor suppressor and regulator of gene expression and proliferation, the exact function of ANP32B is still poorly understood, despite sharing 70% sequence identity and >80% sequence homology with ANP32A. However, in recent years several ANP32B activities connected to the regulation of proliferation of neuronal stem cells, differentiation of leukemic cells and progression from G1 to S phase of the cell cycle have been reported^13,30,31^. Further investigations identified ANP32B as a negative regulator of caspase-3-dependent apoptosis, suggesting that this ANP32 family member is possibly an antagonist of ANP32A in regulating tissue homeostasis^23,32,33^.

More recent studies demonstrated an activity of ANP32B as a histone chaperone, able to recruit histones to certain promoters, thus regulating the transcription of specific genes^34,35^. In addition, several laboratories identified ANP32B as a cellular cofactor or restriction factor involved in regulating viral replication, as demonstrated in the case of foamy virus, adeno-associated virus, henipavirus and influenza virus^36–40^.

Finally, several laboratories showed that ANP32B is necessary for the nucleocytoplasmic transport of specific mRNAs via the uncommon nuclear mRNA export receptor CRM1^7,9,36,37^. Interestingly, for this activity ANP32B must be post-translationally modified by casein kinase II, indicating an additional level of regulatory complexity^41^.

Due to this interesting set of data one could hypothesize that ANP32B can fulfill important functions in cell growth, differentiation, tissue homeostasis and cellular viability *in vivo*, comparable to ANP32A. Unexpectedly, however, the systemic homozygous knockout of Anp32a in mice, like the knockout of Anp32e, did not have any detectable impact on development, behavior, breeding or viability of the affected animals^42,43^. Even combined knockout of Anp32a and Anp32e did not induce a strong phenotype^44^.

In sharp contrast, systemic knockout of Anp32b has a very strong impact during mouse embryogenesis and organ development. In the BALB/c background, homozygous adult animals displayed runtism, pathologies in various organ systems and an unusual clinical chemistry signature^45^. Moreover, in a mixed BALB/c-C57BL/6J background, and associated with a single nucleotide polymorphism, partial perinatal lethality was observed at the end of gestation, while in the pure C57BL/6J background, this lethality was fully dominant. Surviving animals with a BALB/c-C57BL/6J background showed reduced viability and premature aging due to multiple defects in several organs^44^. This may be caused by dysregulated AKT signaling with a subsequent impact on tissue proliferation^46^.

Due to the fatal impact of systemic Anp32b KO on embryonic development^44^, we generated a conditional Anp32b specific KO mouse strain in the C57BL/6 context to investigate whether Anp32b also has an important function in adult animals. This allowed us to analyze whether previously described *in vitro* findings related to ANP32B are also relevant *in vivo*. The resulting phenotype of the Anp32b KO mouse strain was investigated upon Anp32b inactivation in adult animals.

## Results

### Establishment of a Conditional Anp32b KO Mouse Strain

**Figure 1:**
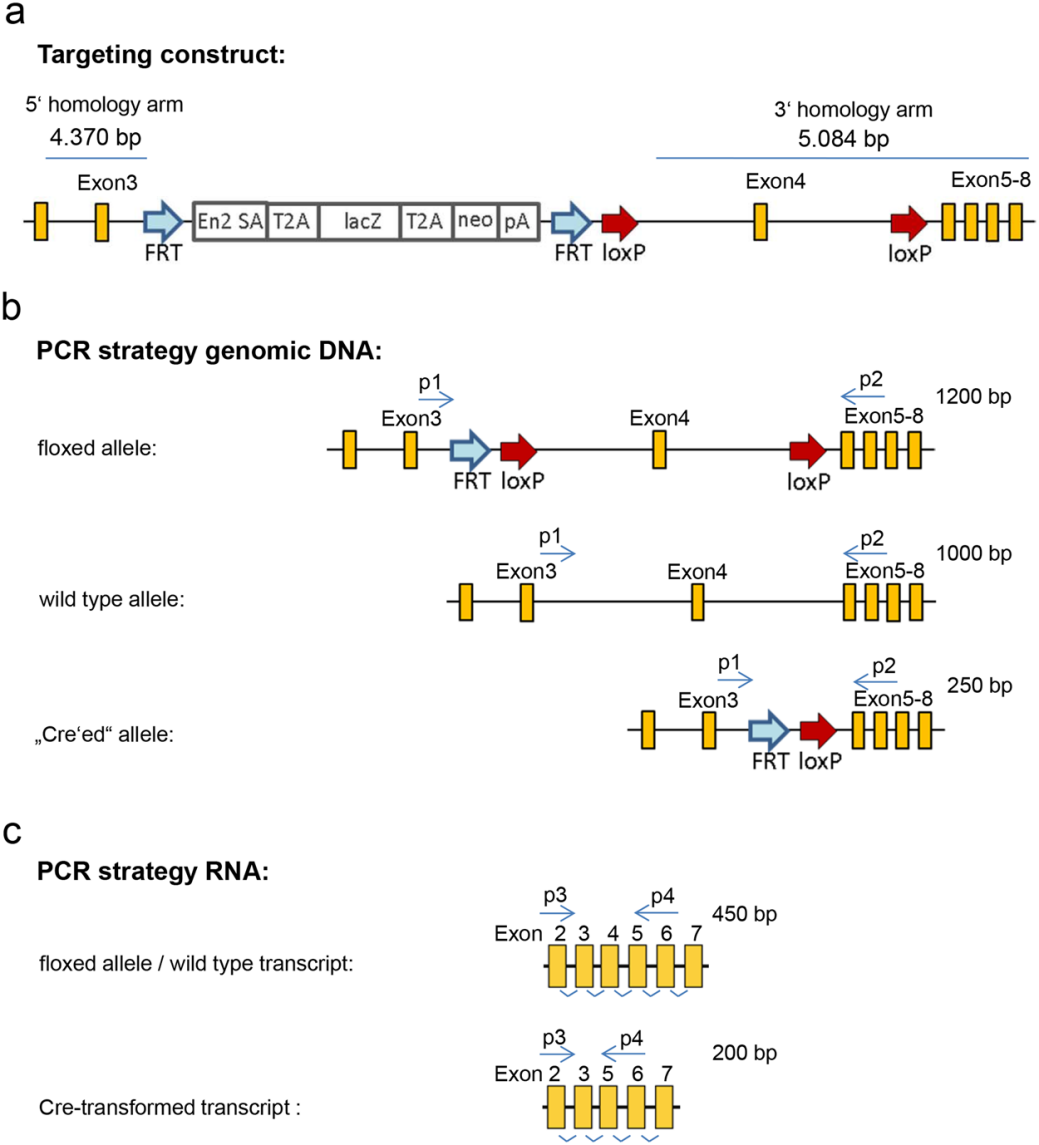
Targeting constructs and PCR strategies. Schematic depiction of **(a)** the original targeting construct and **(b)** targeting variants in the *Anp32b* locus with PCR oligonucleotide primers allowing detection of the respective wild type allele, “floxed” allele and “Cre’ed” allele. **(c)** Targeting variants of *Anp32b* transcripts using PCR primers for detecting *Anp32b* knockout. *FRT*, flippase (Flp) target site; *loxP*, Cre target site; En2 SA, engrailed homeobox 2 splice acceptor; T2A, self-cleaving 2A peptide sequence; lacZ, β-galactosidase reporter gene; neo, neomycin resistance gene; pA, polyadenylation signal.

**Figure 2:**
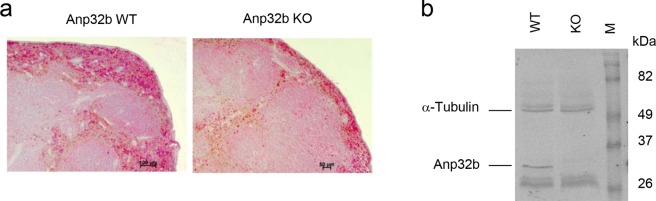
Detection of Anp32b KO in mice. **(a)** Immunohistochemical detection of Anp32b in spleens. Spleen sections from Anp32b KO and wild type (WT) animals were stained with a monoclonal antibody specific for murine Anp32b. **(b)** Detection of Anp32b by Western blot analysis. Single cell suspensions of murine splenocytes from Anp32b KO and WT animals were prepared and crude protein extracts were analyzed using polyclonal ANP32B antiserum.

To generate a Cre-targetable mouse strain, we performed classical BALBc blastocyst microinjection of gene-modified murine C57BL/6N stem cells, where exon 4 of the *Anp32b* gene was flanked by *loxP* sites (EUCOMM JM8.N4 #HEPD0503_1_H01^47^; see Fig. S1). Briefly, speckled chimeric offspring were bred into the C57BL/6 background to screen for germ line transmission. Black offspring were bred with Flp-deleter animals (C57BL/6) to remove the “knockout first cassette” of the targeting construct, including a splice acceptor and polyA site as well as a selection cassette (Neo, LacZ) (Fig. 1a). The resulting strain was crossed with Cre-deleter animals to insert a tamoxifen-inducible Cre cassette (CAG-Cre-ER(T))^48^. Finally, the floxed *Anp32b* allele was bred to homozygosity (Fig. S2a) and the tamoxifen induced *Anp32b* KO was established (by feeding the animals with tamoxifen-containing nutrients) at the chromosomal and transcriptome level in several organs and tissues (Figs S2b,c and S3). To analyze Anp32b KO at the protein level, immuno-histochemical staining of paraffin sections of spleens was performed (Fig. 2a) and confirmed by Anp32b-specific Western blot analyses using crude protein extracts prepared from the animals’ splenocytes (Fig. 2b).

### Analysis of Anp32b KO Animals Under Naïve Conditions

**Figure 3:**
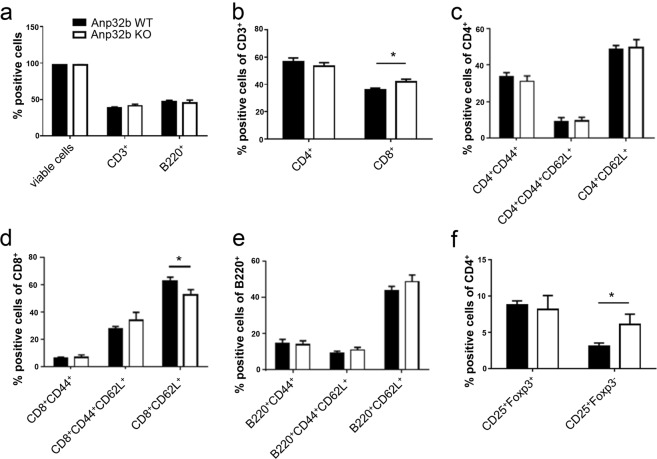
Hematopoietic characterization of Anp32b KO mice. Splenic single cell suspensions from naïve Anp32b KO and WT animals were analyzed for cell counts, viability, T lymphocyte prevalence, activation status and differentiation. **(a)** Splenic T and B cell lymphocytes were analyzed based on CD3 and B220 expression. **(b)** CD3^+^ peripheral T cell subsets were analyzed with respect to their expression of CD4 and CD8 surface molecules. **(c)** The activation status of splenic CD4^+^ or **(d)** CD8^+^ peripheral T cells as well as the activation status of **(e)** B220^+^ B cells was further analyzed with respect to naive and effector memory lymphocyte subsets of the spleen. **(f)** Splenic cells were further analyzed for percentages of CD4^+^CD25^+^Foxp3^+^ T_regs_ and CD4^+^CD25^+^Foxp3^−^ within the CD4^+^ population. Data are mean ± SD. Anp32b WT (n = 4) and Anp32b KO (n = 4). *p < 0.05; Mann-Whitney U test.

Initial investigation of general behavior and fitness, breeding performance, body weight, serology, as well as histopathological analysis of several tissues (i.e. thymus, heart, lung, spleen, intestine, kidney, liver) of Anp32b KO animals did not reveal any obvious phenotypic alterations (see Figs S2 and S3; Table S1). In addition, the abundance of CD68^+^ monocytes and macrophages in the spleens was also not affected (Fig. S8a). Likewise, no impact regarding possible histopathological alterations in femoral bone marrow was observed (Fig. S8b). Analysis of the peripheral B and T lymphocytes and their subsets for their prevalence, differentiation or activation status, did not reveal any obvious strong differences between wild type and KO animals (Fig. 3a–f). In contrast to the abundance of naïve CD8^+^ cells, which were rather reduced in the Anp32b KO mice (Fig. 3d), the overall levels of CD8^+^ cells were slightly increased (Fig. 3b). Furthermore, although compared with wild type mice, the Anp32b KO animals displayed equal amounts of regulatory T cells (T_regs_; CD4^+^CD25^+^Foxp3^+^) and significantly increased numbers of activated CD4^+^CD25^+^ T lymphocytes were detectable (Fig. 3f), indicating low pre-activation of the T cells.

However, using the allogeneic mixed lymphocyte reaction (MLR) assay to assess the activation potential of isolated T cells, no differences between KO and wild type animals were noticed (Fig. S9). Finally, this nearly unaltered phenotype was also observed in cells derived from untreated, and unstressed Anp32b KO mice, with respect to the general apoptosis rate in splenocytes (Fig. S4), the suppressive activity of regulatory T cells (Fig. S5), and, upon *in vitro* activation, the overall proliferation of lymphocytes and the differentiation of T cell subsets (Fig. S6).

### Lowered Th2 Cell Numbers and Increased Th17 Cell Differentiation in Anp32b KO Mice

**Figure 4:**
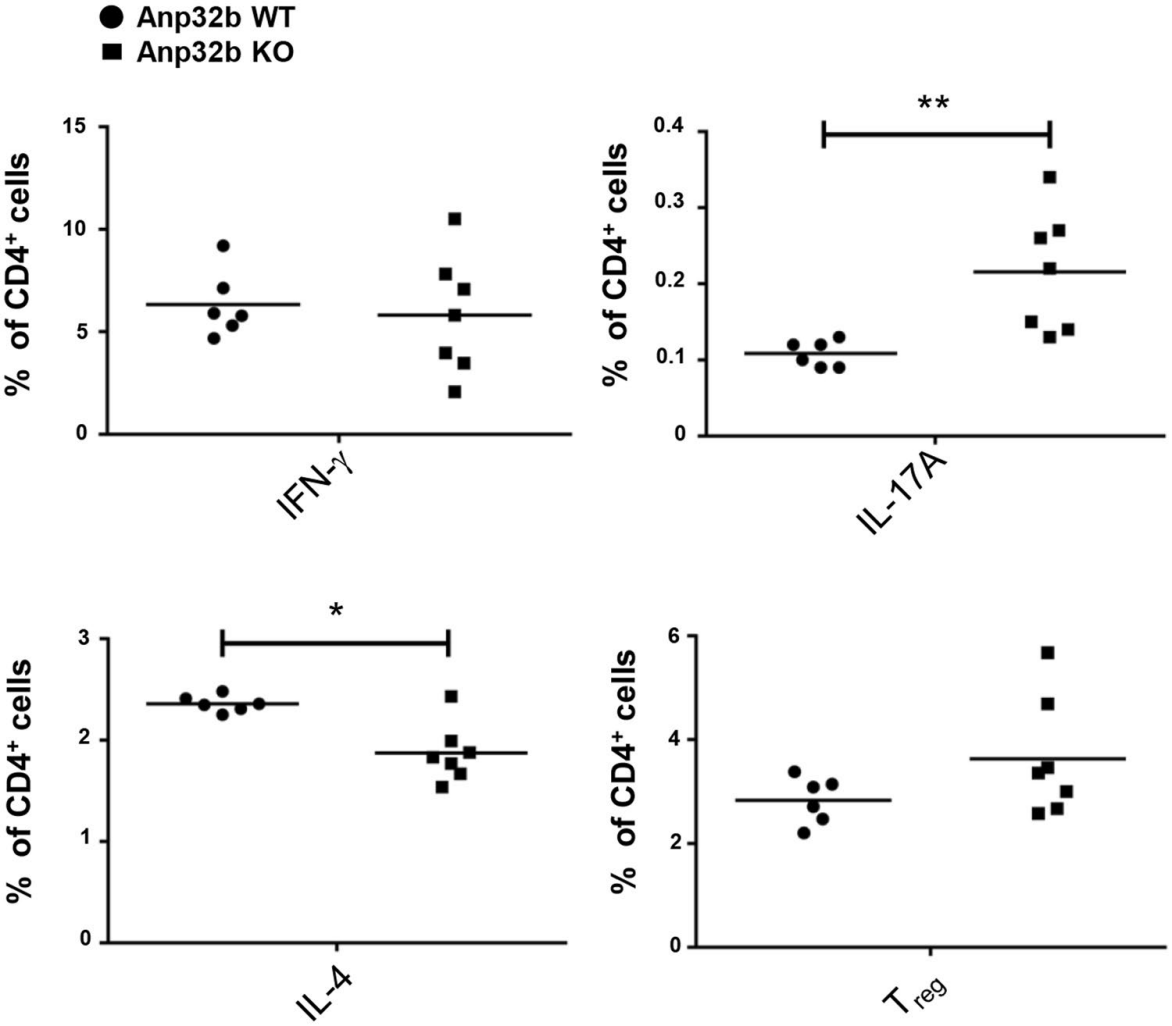
Increased Th17 CD4^+^ T cell population in Anp32b KO mice. Splenic CD4^+^ T cells of Anp32b KO mice and controls were incubated for 4–6 h with 1 mg/ml ionomycin, 20 ng/ml PMA, and 1 mg/ml Brefeldin A (BD GolgiPlug). Cells were stained to determine the percentage of Th1, Th2 and Th17 cells among the CD4^+^ cell population. Anp32b wild type (WT; n = 6) and Anp32b KO (n = 7). Pooled data (mean ± SEM) from two independent experiments are shown. *p < 0.05, **p < 0.01, Mann-Whitney U test was used.

So far, under unstressed, and naïve circumstances no obvious effect on tissue pathology, lymphocyte biology, apoptosis or proliferation of hematopoietic cells was detectable in the Anp32b KO animals. Therefore, to analyze Anp32b KO under immune stimulatory conditions, isolated splenocyte derived CD4^+^ T cells were activated *in vitro* using PMA and ionomycin. The stimulated cells were subsequently incubated with Brefeldin A (BD GolgiPlug) to trap cytokines in the endoplasmic reticulum. Moreover, IFN-γ, IL-4, IL17-A and Foxp3 expression levels were determined to detect Th1, Th2, TH17 or regulatory T cells. The data obtained demonstrated that CD4^+^ T cells derived from Anp32b KO animals were characterized by a significant increase towards Th17-specific immune responses, while at the same time, Th2-specific immune responses were reduced (Fig. 4). However, analysis of the capability of naïve CD4^+^CD62L^+^ splenic cells to proliferate into TH2 specific cells by adding polarizing cytokines, showed no significant impact onto this development per se (data not shown). Furthermore, Anp32b knockout mice displayed normal numbers of Th2 cells within the CD4^+^ T cell population. This indicated that Anp32b deficiency may influence the number of Th17 cells in naïve Anp32b KO animals.

### Strong Enhancement of Experimental Autoimmune Encephalitis in Anp32b KO Mice

**Figure 5:**
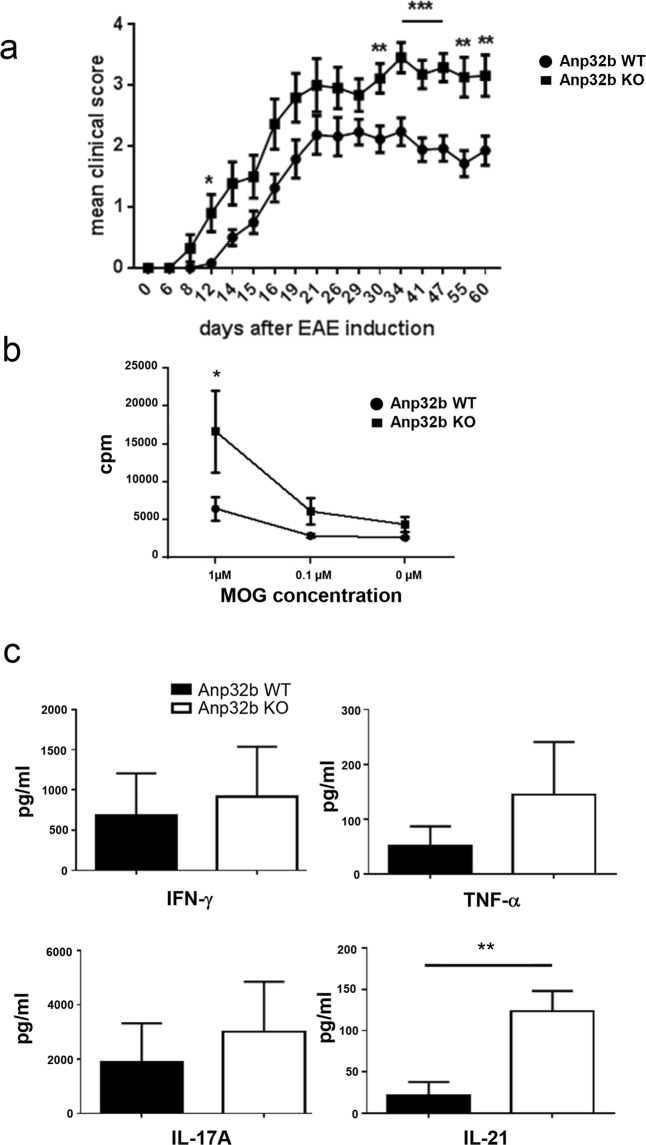
Experimental autoimmune encephalomyelitis (EAE) is enhanced in Anp32b KO mice. **(a)** Representative disease course in Anp32b KO (n = 31) and Anp32b wild type (WT) mice (n = 26) after immunization with MOG peptide emulsified in CFA and enriched with *M. tuberculosis*. Clinical score measurement was performed to indicate the onset, severity and duration of the autoimmune disorder. **(b)** Isolated splenocytes from Anp32b KO and WT animals that had experienced 30 days of EAE were restimulated with MOG peptide at the indicated concentrations. Cell proliferation was subsequently measured after 4 days of restimulation by incorporation of ^[3H]^methyl-thymidine. **(c)** Supernatants from (**b**) were analyzed for expression of IL-17A, TNF-α, and IFN-γ and IL-21. Data in panel (a–c) are mean ± SEM. EAE scoring significances: days 6, 8, 14, 15, 16, 19, 21, 26, 29 (NS); days 12 (p < 0.05); day 30, 55, 60 (p < 0.01) and day 34, 41, 47 (p < 0.001). Pooled data from two independent experiments are shown. *p < 0.05, **p < 0.01, ***p < 0.001, Mann–Whitney U test (a), two-way ANOVA (b), Student t test (c).

Taking into account that Th17/Th1 T cells play a crucial role in autoimmunity, and to unravel a possible role of Anp32b in a more complex and inflammatory immunological environment, we next decided to investigate Anp32b KO animals using the experimental autoimmune encephalitis (EAE) model^49^. Interestingly, immunization of KO mice with myelin oligodendrocyte glycoprotein (MOG)-peptide led to faster appearance of disease symptoms (i.e. paralyses) and to a clearly higher clinical score with ranking significance (Fig. 5a). In line with increased EAE symptoms Anp32b KO mice show more CD45^+^-infiltrates in the brain (Fig. S10).

Furthermore, also the recovery from EAE associated symptoms is inhibited in Anp32b KO mice, as compared to control animals. These data indicated that Anp32b indeed plays a crucial role during the onset, severity and duration of inflammation.

### Enhanced Clinical Symptoms in Anp32b KO Mice Correlate with Depletion of Naïve Effector T Cells, Exhaustion of Lymphocytes and Enhanced Prevalence of Follicular T Helper Cells

**Figure 6:**
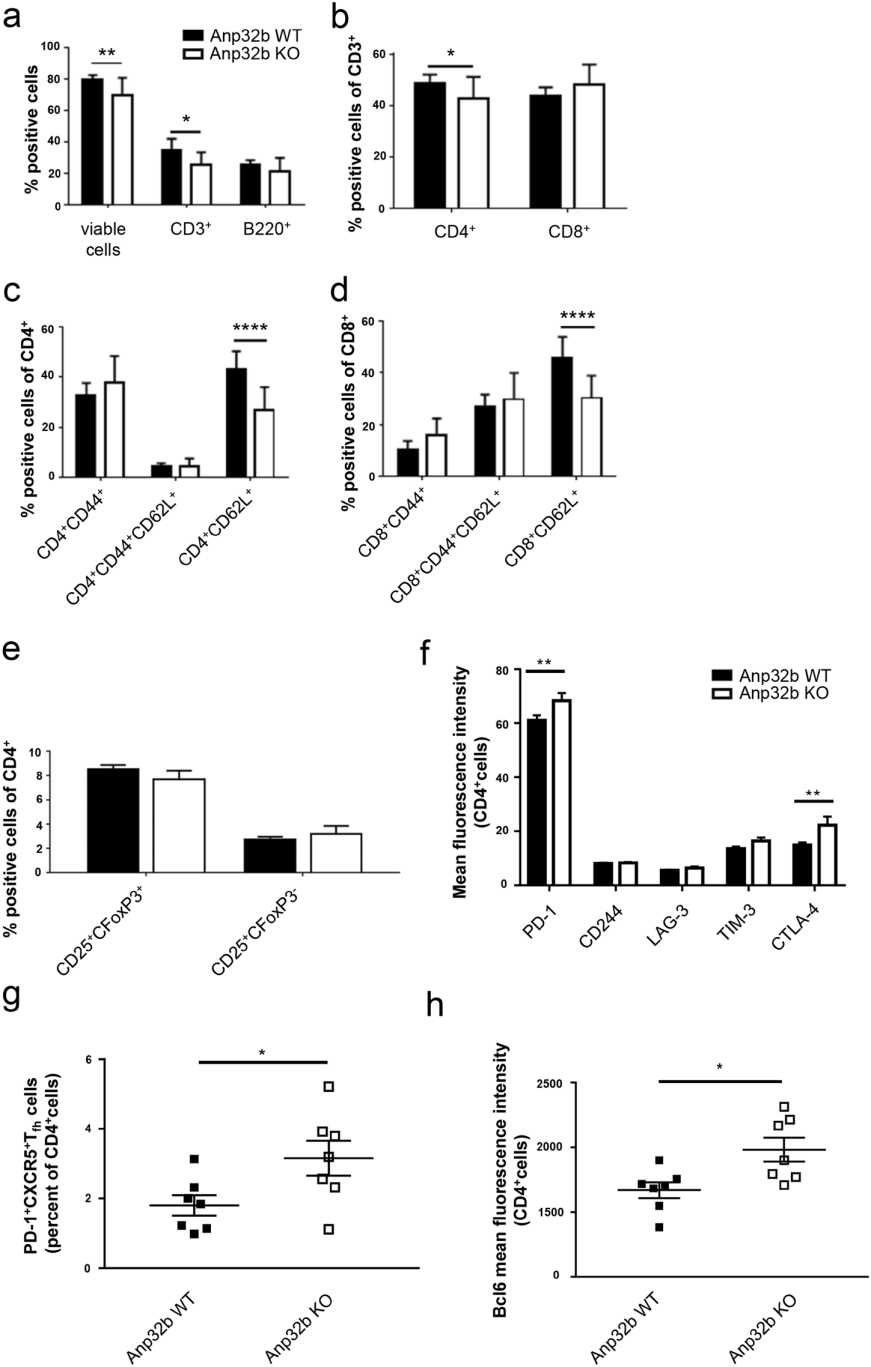
Prevalence and activation status of T cell subsets. Splenocytes of mice that had experienced 60 days of EAE were analyzed as before. **(a)** Splenic T and B cell lymphocytes were analyzed based on CD3 and B220 expression. **(b)** CD3^+^ peripheral T cell subset was analyzed with respect to CD4 and CD8 expression. **(c)** Activation status of splenic CD4^+^ or **(d)** CD8^+^ peripheral T cells were further analyzed with respect to naive T cells and T effector memory cell subsets. **(e)** Splenic cells were analyzed for the percentages of CD25^+^Foxp3^+^ T_regs_ and CD25^+^ Foxp3^−^ within their CD4^+^ population**. (f)** MOG immunized mice were examined for the expression of T cell exhaustion hallmarks. Increased co-expression of multiple inhibitory receptors of Anp32b KO animals in comparison to WT littermates. Lymph node cells were isolated 60 days after MOG immunization. PD-1, CTLA-4, LAG-3, TIM-3 and CD244 expression was analyzed by flow cytometry. **(g)** PD-1^+^CXCR5^+^ among the CD4^+^ T cells from isolated lymph nodes as described above were defined as Tfh cells. Frequencies of Tfh are shown. **(h)** The transcriptional repressor, Bcl-6, that directs Tfh cell differentiation, was determined by flow cytometry within the CD4^+^T cells. Data are mean ± SEM. For (**a–e**): Anp32b WT (n = 12) and Anp32b KO (n = 8); For (**f–h**): Anp32b WT (n = 7) and Anp32b KO (n = 7) or indicated as individual dots in (**h**). *p < 0.05, **p < 0.01, ***p < 0.001 ****p < 0.0001 Mann-Whitney U test.

To further investigate the effects in Anp32b KO animals, splenocytes were isolated from EAE-mice (wild type and KO) at day 30 post EAE induction and then restimulated with MOG-peptide *in vitro*. When compared to control cells, stimulation led to stronger proliferation of Anp32b KO splenocytes (Fig. 5b). Furthermore, secretion of the proinflammatory cytokines TNF-α, INF-γ, IL-17A and IL-21 was strongly enhanced in these cells (Fig. 5c). Subsequent flow cytometry analyses of cells revealed minimally reduced CD3^+^ lymphocyte abundance, but reduced levels of naïve CD4^+^ T cells (CD4^+^/CD62L^+^) and somewhat enhanced numbers of activated CD4^+^CD44^+^ splenocytes (data not shown). However, long-term monitoring of splenic lymphocyte populations for 60 days (Fig. 6) was accompanied by a significant decline in CD4^+^ T lymphocytes, which could be explained by overall reduced CD3^+^ cell numbers. In contrast, CD8^+^ cell numbers were increased in Anp32b KO mice. In addition, CD62L positive naïve CD4^+^ and CD8^+^ T lymphocytes were strongly reduced, whereas activated T lymphocytes were rather increased (Fig. 6a–d). CD4^+^CD25^+^Foxp3^+^ T_reg_ cell numbers are not significantly changed (Fig. 6e). However, it became obvious, that the splenic cells in general showed a more exhausted phenotype indicated by PD-1 and CTLA-4 expression (Fig. 6f). Strikingly, a higher prevalence of follicular T helper cells was observed (Fig. 6g,h), a cell type that have been reported to be involved in autoimmune disorders, as depletion of these cells reduced the pathogenic phenotype^50–52^.

### Enhanced Transcription of Proinflammatory Genes in Anp32b KO Animals

**Figure 7:**
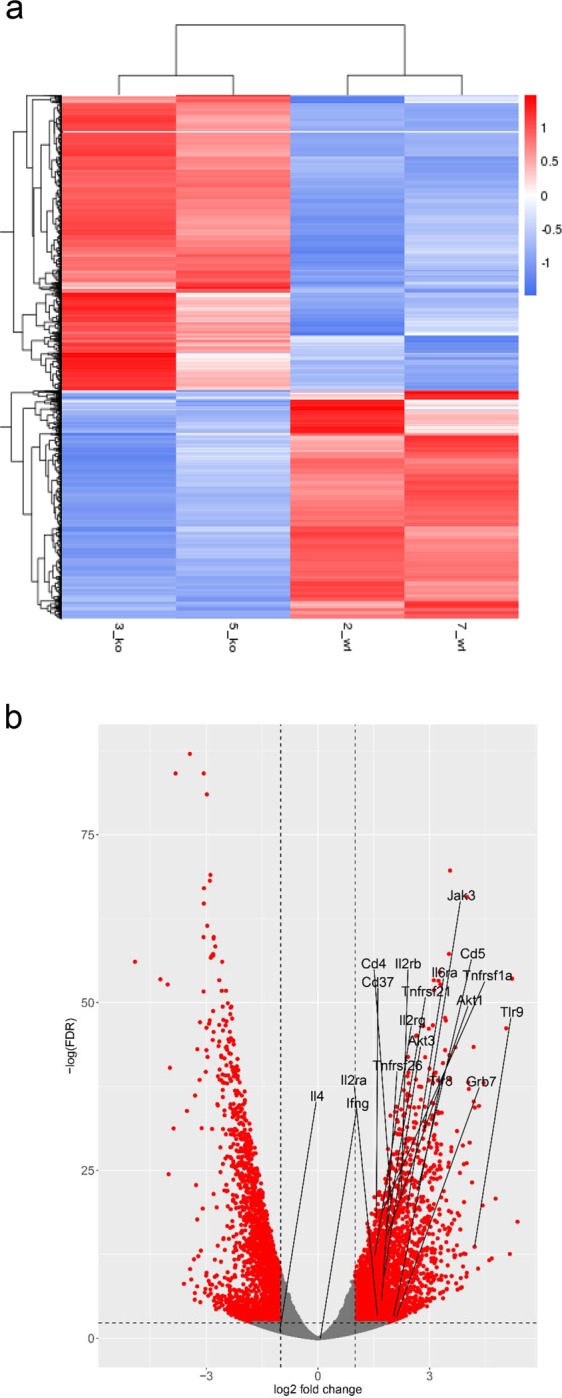
Transcriptome analyses from Anp32b KO splenocytes at onset of EAE. Lymphocytes from Anp32b KO mice and respective control animals were isolated at day 6 of EAE, then total mRNA was isolated and subjected to next generation sequencing. **(a)** Transcript levels relevant for T cell biology were included in a heat map analysis. **(b)** Volcano Plot of significant affected transcripts by Anp32b KO. Special Transcripts relevant for T cell responses are indicated.

**Figure 8:**
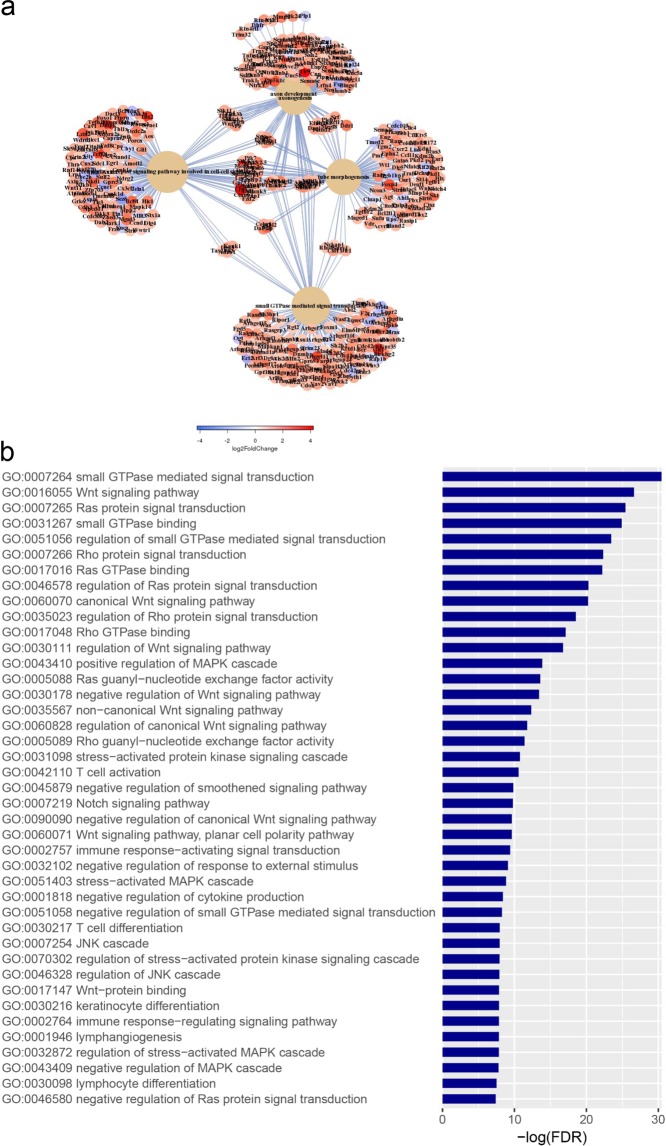
Bioinformatics of gene onthologies. Transcriptome data from Fig. 7 were analyzed for significant regulated cellular pathways. **(a)** Schematic overview of upregulated pathways and ontologies. **(b)** Selection of significantly upregulated pathways (gene onthologies, GO) affecting immune signaling, lymphocyte biology, differentiation and activation.

Next, transcriptome analyses were performed to investigate the impact of Anp32b KO during EAE at the transcriptional level by isolating mRNA from splenocytes of KO and wild type animals (at day 6 after MOG-immunization) and performing next generation sequencing. Results showed, that a large number of transcripts were dysregulated at day 6 post MOG injection in Anp32b KO mice-derived cells (see Fig. 7a). Interestingly, several mRNAs affecting immune responses, particularly transcripts enhancing the activation of effector T cells, were significantly and strongly upregulated in Anp32b KO cells after immunological stress (Fig. 7b). These included key players for T cell activation/expansion, such as IL2, IL2Ra/b, IL6R, CD4, CD5, CD37, TNFr, TLR9, Akt3/1, JAk3, and GRB7. In contrast, messages responsible for the induction of TH2 immune responses, for example IL-4, were clearly reduced. Bioinformatics of the affected transcripts revealed an impact of Anp32B knock out on huge numbers of pathways. Significantly downregulated gene onthologies (GO) (126 GOs, FDR < 0.01) included mitochondrial pathways involved in energy metabolism, pathways involved in ribosomal protein synthesis and overall DNA replication (data not shown). Significantly upregulated gene onthologies (GO) (540 GOs, FDR < 0.01) (Fig. 8a) included pathways for cell growth, tissue and neuronal development and regulation of cell death. In addition, further pathways frequently affect cellular signaling and are involved in various aspects of the regulation of immune responses, including lymphocyte differentiation and activation (Fig. 8b).

Thus, the observed altered transcription of proinflammatory mRNAs as well as transcripts involved in immune signaling and lymphocyte biology correlated well with the enhanced pathogenic phenotype observed in MOG-immunized Anp32b KO animals.

## Discussion

Recently, several *in vitro* studies demonstrated the involvement of the acidic protein rich in leucines ANP32B in the regulation of cell proliferation, apoptosis, cell cycle progression and gene expression at the epigenetic and posttranscriptional level^7,9,10,13,24,31,32,41^. In addition, involvement of ANP32B in viral infection, acting either as a critical cellular cofactor or as restriction factor, has been reported for various viruses^36–40^. However, most of these data were derived from *in vitro* studies. A systemic KO of Anp32b in mice has been previously shown to result in embryonic lethality^44^. Here, we succeeded in generating a conditional systemic Anp32b KO in adult animals and, hence, were able to show that Anp32b is a modulator of adaptive immune responses.

By targeting the murine *Anp32b* gene with *loxP* sites for Cre-dependent recombination, and using a tamoxifen inducible recombinase expression cassette (CAG-Cre-ER(T))^48^ all cells should be affected in Anp32b KO animals upon feeding with tamoxifen. This experimental strategy led to no obvious adverse effects in adult and immunologically unchallenged Anp32b KO animals, such as overall viability and fitness, breeding behavior, organ development, hematopoiesis, or clinical chemistry parameters. This rather wild type-like phenotype of Anp32b KO largely reflected the previously reported observations in mice, where the Anp32 family members a and e were inactivated^42,43^.

Since ANP32B has been previously correlated with expression of immunologically relevant proteins in lymphocytes and dendritic cells^7,41^, we compared the stimulatory capacity of splenocytes derived from Anp32b KO mice to control animals. Our data revealed an impact of Anp32b KO on the ratio of Th1/Th17 versus Th2 immune responses. *In vitro* differentiation analyses showed normal development of these cell types, thus Th2 immune responses per se are not affected, but this has to be invested in a further study. However, because of the strong Th17 response in the Anp32b KO cells the respective mice were further analyzed using an EAE model, as these cells have been correlated with autoimmune reactions. Interestingly, knockout of Anp32b led to earlier induction, stronger progression and longer extension of the autoimmune pathology. This could be correlated with hyperactivation of splenic lymphocytes at mRNA levels, protein expression, activation and exhaustion status as well as cellular differentiation levels.

The impressive and unexpected dysregulation of immunologically relevant transcript expression resulted in stronger activation of Th17 immune responses, as well as weaker Th2 responses. Furthermore, the strong upregulation of follicular Th cells in Anp32b KO mice suffering from an EAE furthermore indicate a substantial dysregulation of immunologic pathways in these animals and an involvement of the Anp32b protein in fine tuning of this complex pathways. The hyperactivated immune status, the strengthened Th17 response, especially the reduction of naïve lymphocytes and the strong exhaustion status of the T cells as well as the increased presence of Thf cells in Anp32b KO mice suffering from EAE furthermore would explain the strong progression of the autoimmune process in these animals as reflected by literature^53,54^.

In summary, we provide evidence that Anp32b is necessary to find a balance in the interplay of m-RNA expression, cellular differentiation and exhaustion status of T lymphocytes and the regulation of adequate immune responses. These findings may have an important impact on the development of autoimmune disorders, and unraveling the underlying mechanisms could lead to a more detailed insight into this complex dysregulated network and may help to find new drugable targets to treat autoimmune disorders in the future.

## Methods

### Animal studies

All animal procedures were approved by the Hamburg State Authority according to the German Animal Welfare Act (permission number 132/13). Mice were maintained in specific pathogen-free conditions at the Heinrich Pette Institute – Leibniz Institute for Experimental Virology Hamburg, or at the Department of Immune Modulation, Universitätsklinikum Erlangen.

### Generation of conditional knockout mice

Embryonic stem cell clones derived from C57BL/6 mice for conditional Anp32b knockout (exon 4 of *Anp32b* gene was flanked with loxP sites, JM8.N4 #HEPD0503_1_H01) were obtained from the EUCOMM – International Knockout Mouse Consortium^47^. Briefly, stem cells were characterized by Southern blotting and PCR methods and microinjected into BALBc blastocysts. Speckled chimeric offspring were back-bred into the C57BL/6 background. Deletion of the selection cassettes was performed by breeding the animals with Flp-deleters (C57BL/6). The resulting mouse strain was crossed with the final Cre-deleter strain (C57BL/6) to insert a heterozygous tamoxifen-inducible Cre expression cassette (CAG-Cre-ER(T))^48^ and then again bred homozygous for the “floxed” *Anp32b* gene. Maintenance breeding was done with male homozygous Anp32b “floxed”/heterozygous Cre animals and female homozygous Anp32b “floxed” animals. According to Mendelian distribution, half of the offspring, homozygous for “floxed” *Anp32b* gene, were positive for Cre and therefore could be used for *Anp32b* knockout induction. Tamoxifen (4-OHT) induced knockout was performed by administering 4-OHT containing nutrients (400 mg/kg, LASCRdiet CreActive TAM400, Genobios) for up to 4 weeks. For phenotyping, Cre positive and Cre negative sister animals were fed in parallele and Cre negative animals served as negative controls (wild type).

### PCR and southern blotting

Southern blotting was performed as described previously^55^. PCR was performed with 0.5 µg genomic DNA and Phusion® High-Fidelity DNA Polymerase KIT (New England Biolabs) according to the manufacturer’s instructions: Generally, 2 cycles at 94 °C followed by 35 cycles of 10 sec at 98 °C, 30 sec at 63 °C and 45 sec at 72 °C and finalized by 5 min at 72 °C.

### Genotyping

Genomic DNA from ear-tag biopsies was isolated (DirectPCR Lysis Reagent, VWR) and subjected to PCR analyses using the following DNA oligonucleotide primers: Anp32b allele: P1, 5′-ggttttgtttaattttgggagagcactaaactta-3′; P2, 5′-gaaaatgatgaactctaagcacagaaaggattct-3′. Cre allele: Cre-for, 5′-ctctagagcctctgctaacc-3′; Cre-rev, 5′-cctggcgatccctgaacatgtcc-3′. PCR products were subsequently analyzed by gel electrophoresis. Anp32b knockout at the RNA level was analyzed by PCR using cDNA and the following DNA oligonucleotides: P3, 5′-cttgagctcagtgaaaatagaatcttcggag-3′; P4, 5′-cctccccgctgacttcatcctcatcatc-3′.

### Paraffin sections and immunohistochemical staining

Immunohistochemical analyses were performed as described previously^56,57^. Briefly, organs were fixed with 2% buffered paraformaldehyde for at least 2 d at 4 °C. Femora were decalcified in 10% (w/v) EDTA in PBS for 4 d. Organs were dehydrated through serial ethanol exposures, embedded in paraffin wax and sections of 4 µm thickness were prepared using a Thermo Fisher microtome. Sections were rehydrated and treated for five min with Fast Enzyme (Cytomed) at room temperature for antigen retrieval. After three washes (5 min each) in TBS buffer, the primary anti-ANP32B antibody (ABIN872630; Antibodies Online) diluted 1:100 in Dako antibody diluent (Dako) was applied at room temperature in a moist chamber. Rabbit negative control antibodies (Dako X0903) served as an isotype control. After three careful washes (3 × 5 min) in TBS, antibody binding sites were located with the Dako REAL detection system alkaline Phosphatase (K5005). A light hemalumn stain was used for counterstaining. Sections were dehydrated and mounted in Eukitt (Kindler).

### Brain histology

Brains of mice were harvested at day 60 of EAE, fixed in liquid nitrogen, and stored at −80 °C. Organs were embedded in Tissue-Tek (Sakura), and 5-μm sections were made using a cryotome (Kryocut CM 2000; Leica). Immunohistological staining was performed using an immunoperoxidase detection system in a humid incubation chamber. Acetone-fixed sections were incubated in PBS, and endogenous peroxidase activity was blocked by incubating sections in 3% H_2_O_2_. Sections were incubated with a primary anti-CD45 Ab (clone 30G12; provided by L. Sorokin, Lund University, Lund, Sweden). The primary Ab was detected by a streptavidin HRP–coupled goat ant-rat IgG Ab (Biocare Medical). To visualize Ags, sections were incubated with AEC Chromogen Substrate (Vector Laboratories). Sections were stained with hematoxylin (Sigma-Aldrich), mounted in Aquatex (Merck), covered with a coverslip, and examined by light microscopy (Leica).

### Bone marrow histology

For bone marrow histology mouse femurs were isolated and placed in 10% buffered formalin. After decalcification the tissues were embedded in paraffin, sectioned, and stained using standard Wright-Giemsa protocol. Histology images were taken with a Zeiss Axioplan 2 microscope (Carl Zeiss).

### Isolation of mouse splenocytes

Isolated spleens were disrupted in ice cold buffer (RoboSep buffer, Stemcell Technologies). Cell suspensions were prepared by percolation of the spleen fragments using Cell Strainers (BD Falcon). Immediately, cells were harvested by centrifugation (150 × g, 5 min, 4 °C), suspended in suitable medium and cultured at ambient conditions or used directly for FACS analysis.

### Protein analyses

Western blot analysis was performed as described previously^41,58^. Briefly, crude extracts were prepared by lysis of the cells with lysisbuffer A (0.1% NP40, 150 mM NaCl, 50 mM Hepes at pH 7.3, protease inhibitors) followed by denaturation in SDS-buffer (65 mM Tris-HCl pH 6.8, 2% SDS, 10% glycerol, 1.5% DTT). SDS-PAGE was carried out using 13% polyacrylate-gels and Western blot analysis was performed with polyclonal rabbit antiserum specific for ANP32B (described in^7^).

### Annexin V assay

For the apoptosis assay of primary mouse cells, cells were stained with Annexin V APC Apoptosis Detection Kit (Biolegend) according to manufacturer’s instructions.

### Blood serum analyses

Levels of creatinine (enzymatic, mg/dL), glucose, alkaline phosphatase (ALP, (IU/L), alanine aminotransferase (ALT, (IU/L), total bilirubin (TBIL, mg/dL) and lactate dehydrogenase (LDH, IU/L) were determined using the UniCel DxC 800 Synchron Clinical System (Beckman Coulter International).

### Transcriptome analysis

Total cellular RNA was isolated according to the manufacturer’s protocol using TRIzol reagent (Invitrogen). cDNA libraries were prepared with NEBNext Ultra II DNA Library Prep Kit for Illumina and NEBNext Multiplex Oligonucleotides for Illumina (New England Biolabs) according to manufacturer’s instructions. Sequencing was performed on the Illumina NextSeq system. Sequencing reads were aligned against the mouse reference assembly (GRCm38.p5) and quantified using STAR (v2.5.2b)^59^. Based on these counts, statistical analysis of differential expression was carried out with DESeq. 2 (v1.14.0)^60^.

### Fluorescence activated cell sorting

Splenic single-cell suspensions were incubated with fluorochrome-conjugated Abs (FITC, PE, PECy7, PerCPCy5.5, allophycocyanin, APCCy7) in PBS for 30 min at 4 °C. For intracellular staining, the eBioscience Foxp3/Transcription Factor Staining Buffer Set was used, according to the manufacturer’s instructions. Stained cells were analyzed using FACSCanto II devices (BD Biosciences) and FCS Express5Flow software (DeNovo). Viable lymphocytes were gated based on forward scatter and side scatter prior to further gating of fluorescent-labeled populations. The following Abs were used: anti-CD4–APC/Fire™750-RM4-5, anti-CD3-BV421 17A2, anti-B220–APC RA3-6B2 7D4, anti-CD44-PerCPCy5.5 IM7, anti-CD11b–FITC M1/70, anti-IL-17-AF614 TC11-18H10.1, anti-CD19- PerCPCy5.5 6D5, anti-CD25–APCCy7 PC61, anti-mouse Ki-67-AF488 16A8, viability staining solution-PerCP 7-AAD, anti-PD-1-APC RMP1-30, anti-CD244-FITC m2B$(B6)458.1, anti-LAG-3-PE C9B7W, anti-TIM-3-PerCP B8.2C12, anti-CTLA-4_PECy7 UC104B9, anti-CXCR5-bio L138D7, Streptavidin-BV421, anti-Bcl-6-PA IG191E/A8 (all from Biolegend). Anti-CD8a–PerCP 53-6.7, anti-CD25–FITC 7D4, anti-CD62L-APC MEL-14, anti-IFNγ-PECy7 XMG1.2, anti-IL-4- PE 11B11 (RUO) (all from BD Biosciences) and anti-Foxp3–PE FJK-16s (all from eBioscience).

### Mixed lymphocyte reaction

Bone marrow-derived DCs were generated from BALB/c mice, as described elsewhere^61^. At day 9, bone marrow–DC cultures were matured with LPS overnight or were left unstimulated. A total of 4 × 10^6^ allogeneic splenic cells, from Anp32b wild type or Anp32b KO mice were incubated with differentially stimulated DCs in a 96-well flat-bottom plate (Falcon) for 72 h at 37 °C and 5% CO2. Then, 1 μCi/well [^3^H]-methyl-thymidine (Amersham Biosciences) was added, and cells were incubated for an additional 16 h. The plates were harvested with an IH-110 harvester (InnoTech), and filters were counted in a 1450 Microplate Counter (Wallac) to detect incorporated radioactivity as readout for proliferation.

### Induction of EAE

To induce EAE, mice were immunized (s.c.) with 50 μg MOG35–55 peptide (Charite) emulsified in CFA, which was enriched with 10 μg/ml Mycobacterium tuberculosis (H37Ra; Difco/BD). Additionally, 200 ng of pertussis toxin (List/Quadratec) was injected i.p. at days 0 and 2 of EAE. Scoring of EAE symptoms was performed as follows: 0, no disease; 1, tail weakness; 2, paraparesis; 3, paraplegia; 4, paraplegia with forelimb weakness; and 5, moribund or dead animals.

### MOG restimulation

At day 30 or day 60 after MOG injection, splenic cells from mice were harvested, and 4 × 10^5^ total spleen cells or purified CD4^+^ splenic cells were incubated with different concentrations of MOG peptide and/or 50 U/ml IL-2 (Proleukin) in 200 μl RPMI medium supplemented with 10% FCS, 100 U/ml penicillin, 100 μg/ml streptomycin, 2 mM L-glutamine, and 50 μM 2-ME (all additives were from Sigma-Aldrich) per well in a 96-well tissue culture plate for 72 h at 37 °C and 5% CO2. Then, 0.1 μCi/well ^[3H]^methyl-thymidine (Hartmann Analytic) was added and cells were incubated for an additional 12 h. Subsequently, the plates were harvested with an IH-110 harvester (InnoTech), and filters were counted in a 1450 Microplate Counter (Wallac) to detect incorporated radioactivity as readout for proliferation. Supernatants from all restimulated cultures were harvested to determine *ex vivo* cytokine production.

### Cytokine quantification/ELISA

To determine cytokine production in supernatants of MOG-restimulated DCs in T cell co-cultures, a commercially Cytometric Bead Array (BD Biosciences) was used to detect IL-17A, IFN-γ, IL-21, and TNF-α, according to the manufacturer’s instructions.

### Stimulation of CD4^+^CD62L^+^ T Cells

CD4^+^CD62L^+^ naïve T cells were obtained from spleen and lymph node of naive mice using a CD4^+^CD62L^+^ T Cell Isolation Kit II (Miltenyi Biotec), according to the manufacturer’s instructions. Cells were stimulated with 1 μg/ml ionomycin and 20 ng/ml PMA, as well as 1 μg/ml Brefeldin A (BD GolgiPlug), and incubated for 6 h at 37 °C and 5% CO_2_ and analyzed for the expression of intracellular cytokines IL-17A, IFN-γ, IL-4 and Foxp3.

### Suppression assay

For the suppression assay, T_eff_ and T_regs_ were cultured on feeder cells derived from RAG1^−/−^ mice. These cells were treated with Low-Tox^®^-M Rabbit Complement (Cedarlane) and an anti-mouse CD90.2 antibody (30-H12; BioLegend). Afterwards, 5 × 10^4^ feeder cells were seeded into 96-well roundbottom plates (BD Falcon). CD4^+^CD25^−^ Teff and CD4^+^CD25^+^ Tregs were isolated from naive Anp32b KO and wild type mice or EAE treated KO an wild type animals (day 60) using the mouse CD4^+^CD25^+^ Regulatory T cell Isolation Kit (Miltenyi Biotec) according to the manufacturer’s instructions. Purity of sorted cells was assessed by FACS directly after isolation and revealed ≥92%. T_eff_ were labeled with 0.75 µM CellTraceTM Violett (CellTraceTM Violett Cell Proliferation Kit; molecular probes by life technologiesTM) per 10^6^ cells and 5 × 10^4^ labeled T_eff_ per well were added to the RAG1^−/−^ feeder cells. Finally, T_regs_ were plated onto these cells in titrated numbers and co-cultures were stimulated using 0.25 mg/ml of an anti-mouse CD3ε antibody (145-2C11; BioLegend) and incubated for 72 h before percentage of proliferating cells was determined by FACS. Results were analyzed using FCS Express Flow Cytometry Software (*De Novo*).

### Statistics

Results are expressed as mean and SEM, unless stated otherwise in the figure legends. A two-tailed Student t test or and two-way ANOVA with the Bonferroni posttest (for multiple groups) were used to determine statistical significance, unless stated otherwise in the figure legends. A nonparametric Mann–Whitney U test was used to analyze clinical EAE scoring. All statistical tests were two-sided and differences with p values of *p < 0.05, **p < 0.01 and ***p < 0.001 were considered statistically significant. Statistical analyses were performed with Excel or Prism 4.03 or 5.0 (GraphPad).

## Supplementary information

Chemnitz et al., Supplementary Information

## Data Availability

The datasets generated during and/or analysed during the current study are available from the corresponding author on reasonable request.
