# Supplementary material for: The acidic protein rich in leucines Anp32b is an immunomodulator of inflammation in mice
Source: Sci Rep. 2019 Mar 19;9:4853. doi: 10.1038/s41598-019-41269-z (PMC6424966; doi:10.1038/s41598-019-41269-z)
Supplement: Supplementary file 1 — Chemnitz et al., Supplementary Information [file 41598_2019_41269_MOESM1_ESM.pdf]

The acidic protein rich in leucines Anp32b is an immunomodulator of inflammation in mice

Supplementary Information

Jan Chemnitz[a,1], Dorothea Pieper[a], Lena Stich[b], Udo Schumacher[c], Stefan Balabanov[d], Michael Spohn[a], Adam Grundhoff[a], Alexander Steinkasserer[b], Joachim Hauber[a,e] and Elisabeth Zinser[b,1]

[a]Heinrich Pette Institute – Leibniz Institute for Experimental Virology, Martinistrasse 52, 20251 Hamburg, Germany; [b]Department of Immune Modulation, University Hospital Erlangen, Hartmannstrasse 14, 91052 Erlangen, Germany; [c]Center for Experimental Medicine, Department of Anatomy and Experimental Morphology, University Medical Center Hamburg-Eppendorf, Martinistrasse 52, 20246 Hamburg, Germany; [d]Division of Hematology, University Hospital Zurich, Rämistrasse 100, CH-8091 Zurich, Switzerland; [e]German Center for Infection Research (DZIF), Partner site Hamburg, Germany.

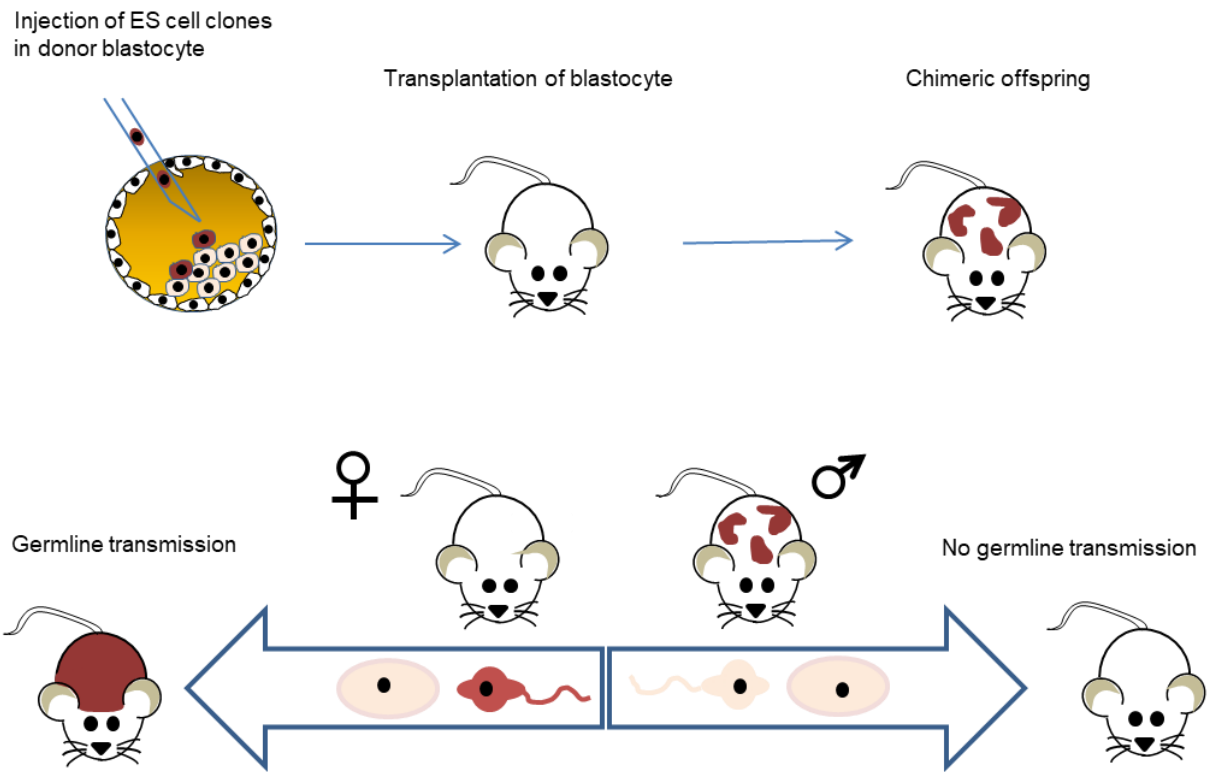

Fig. S1. Establishment of conditional Anp32b KO mice. Gene modified murine C57BL/6N stem cells (carrying a floxed *Anp32b* exon 4) were microinjected into blastocysts of BALBc mice. Speckled chimeric offspring were bred into the C57BL/6 background to screen for germ line transmission (black offspring, indicated in red).

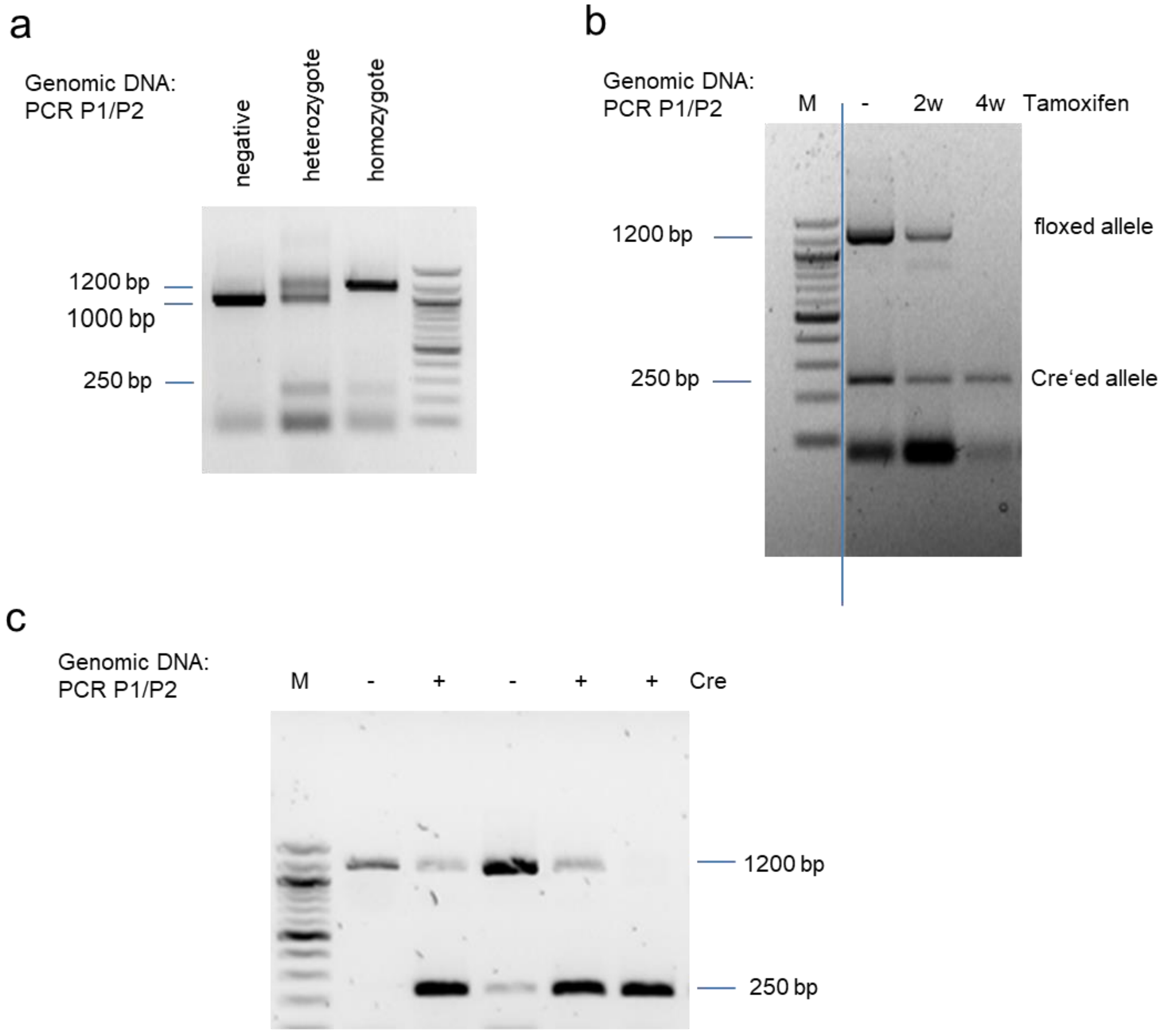

Fig. S2. Homozygous knockout of Anp32b in mice. (a) PCR based detection of wild type and “floxed” *Anp32b* alleles. Genomic DNA from ear-tag biopsies were subjected to PCR analysis with primer pair P1/P2 (see Fig. 1) **(b)** Detection of time dependent tamoxifen-induced recombination of “floxed” *Anp32b* gene. Genomic DNA from PBMC from Anp32b KO mice fed with tamoxifen containing nutrients was isolated at the indicated time points and subjected to PCR analysis (P1/P2). The grouping of gels from different parts of the same gel is indicated by a separating blue line. **(c)** Tamoxifen dependent knock out of the *Anp32b* gene in selected animals. Data show reliable *Anp32b* knock out (w; weeks of tamoxifen treatment). M: DNA marker 100 bp ladder.

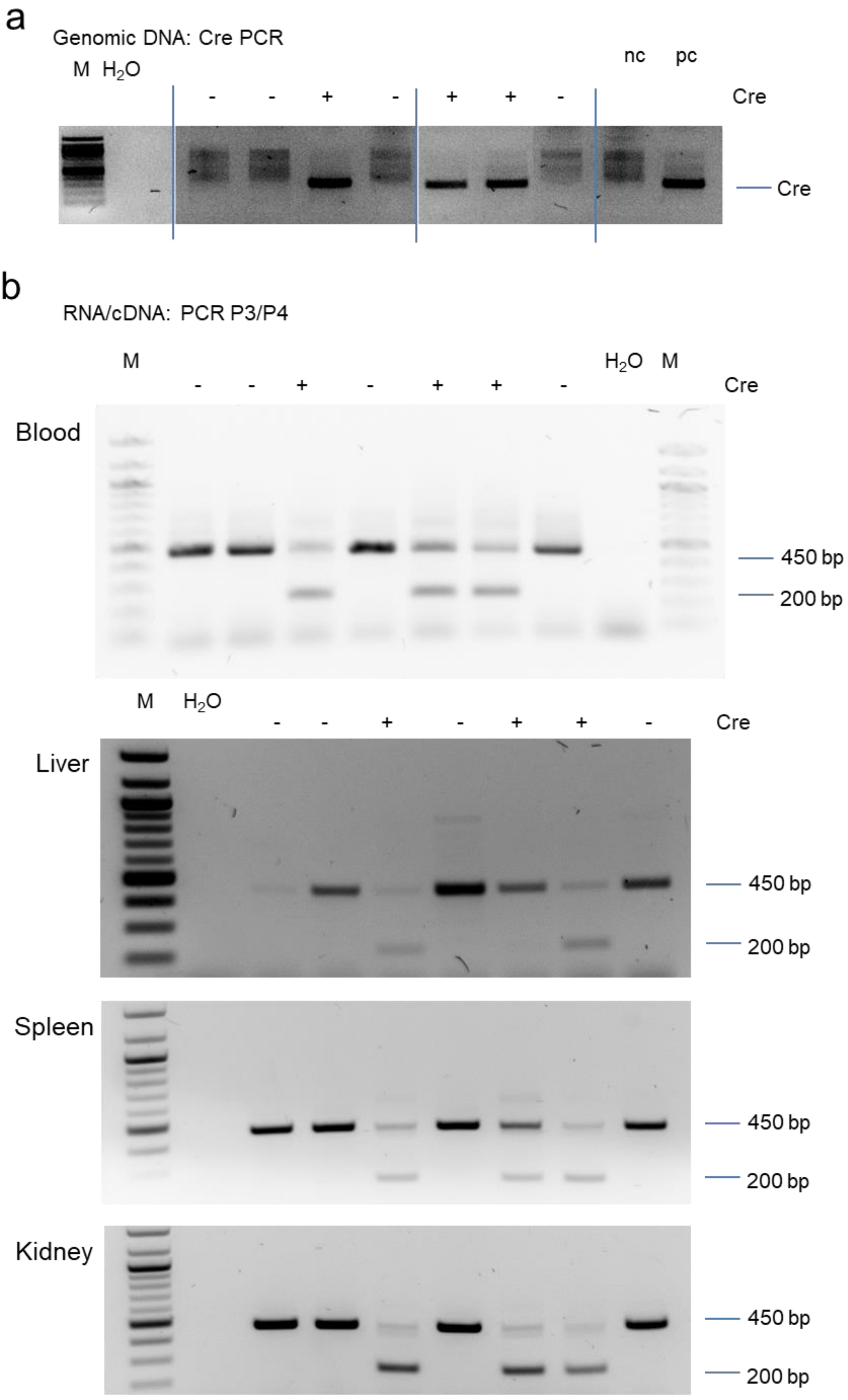
a
Genomic DNA: Cre PCR
M H2O
nc pc
- - + - + + -
Cre
Cre
b
RNA/cDNA: PCR P3/P4
M H2O M
- - + - + + -
Cre
Blood
450 bp
200 bp
M H2O
- - + - + + -
Cre
Liver
450 bp
200 bp
Spleen
450 bp
200 bp
Kidney
450 bp
200 bp

Fig. S3. Cre-dependent abrogation of *Anp32b* mRNA accumulation. (a) PCR-based detection of Cre-specific genomic sequences. Genomic DNA was prepared from ear-tag biopsies from several sister animals. The grouping of gels from different parts of the same gel is indicated by a separating blue line. **(b)** Detection of *Anp32b* mRNA in the indicated tissues. Animals were fed with tamoxifen for at least 4 weeks followed by preparation of various organs. Single cell suspensions were prepared and total RNA was isolated, reversely transcribed and subjected to PCR using P3/P4 primers (see Fig. 1). M: DNA marker 100 bp ladder; H_2O: water control; nc: negative control; pc: positive control.

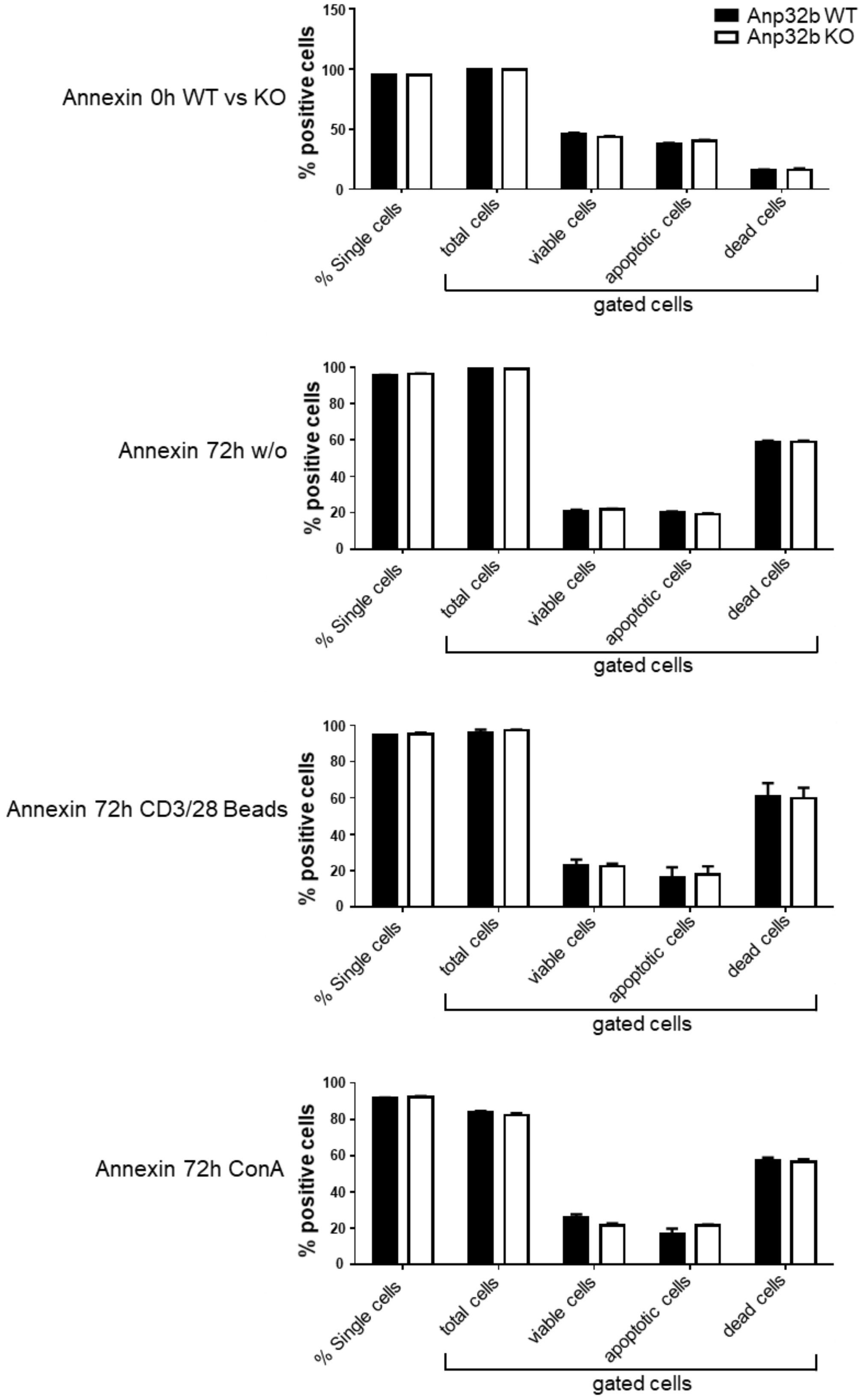

Fig. S4. Apoptosis in naïve Anp32b KO splenocytes. Splenocytes from naïve wild type (WT) and Anp32b KO mice were isolated and single cell suspensions were directly

analyzed (top panel), or cultured for 72 hours without further treatment (w/o), treated for 72 hours with CD3/CD28 beads, or treated for 72 hours with Concanavalin A (ConA) prior to direct analysis for surface expression of Annexin V as a measurement for the onset of apoptosis. Data are mean ± SEM. Anp32b WT (n = 4) and Anp32b KO (n = 4).

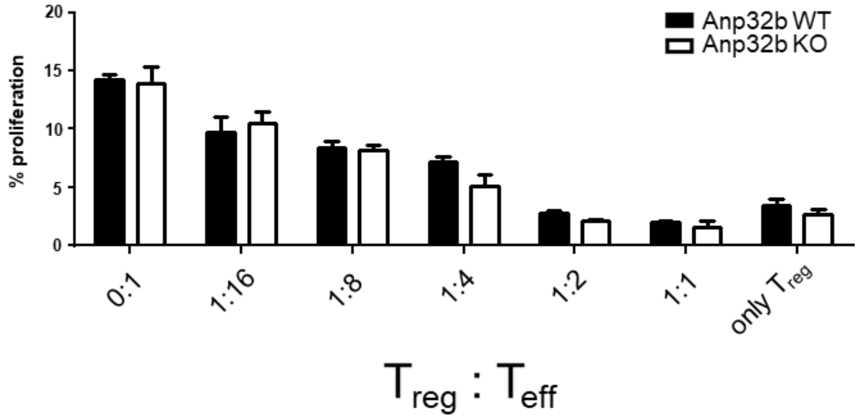

Fig. S5. Suppressor activity of naïve regulatory T cells. Splenic regulatory T cells from naïve WT and Anp32b KO mice were isolated and single cell suspensions were subjected to an *in vitro* suppression assay with indicated T_{reg}:T_{eff} ratios to investigate T_{reg} functionality. Data are mean ± SEM. Anp32b WT (n = 4) and Anp32b KO (n = 4).

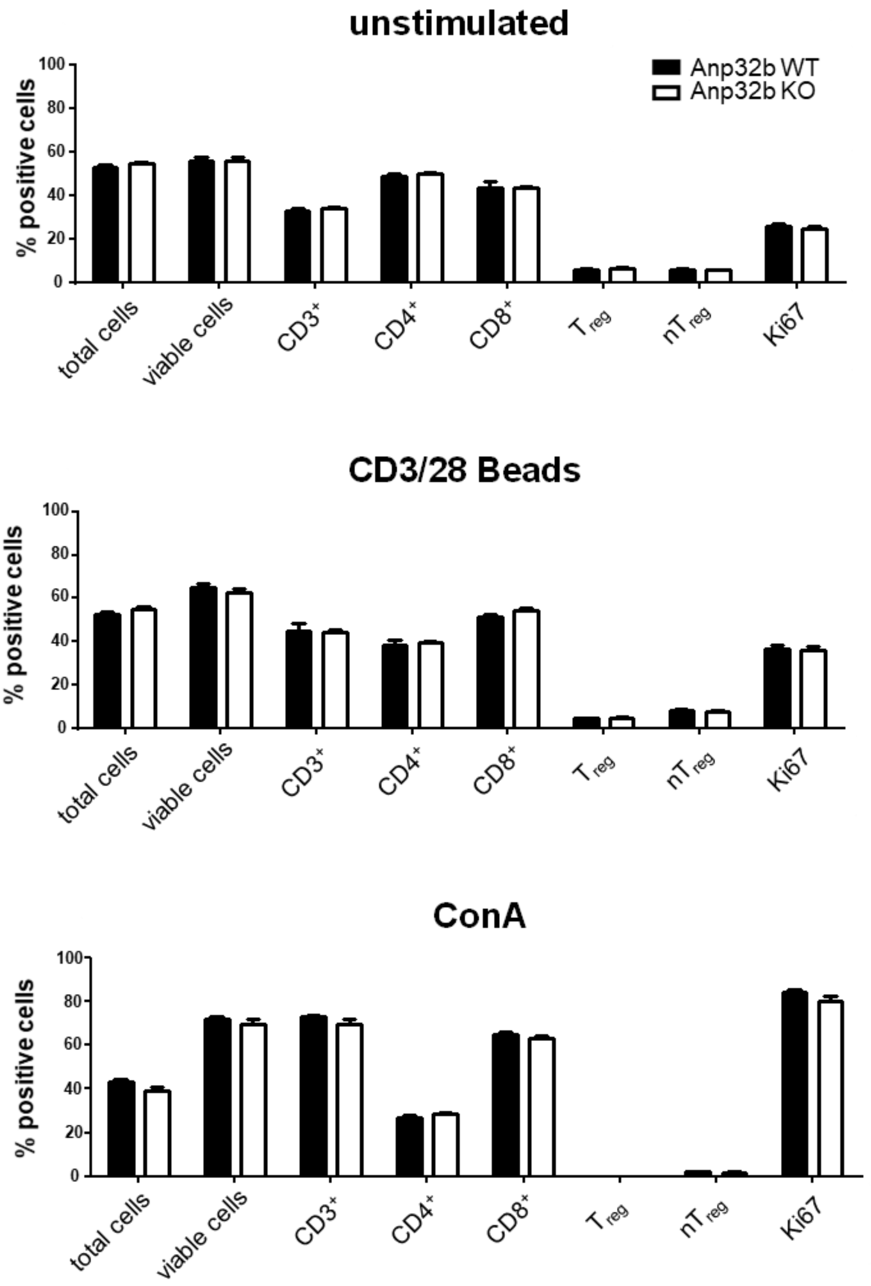

Fig. S6. Cell counts, viability, proliferation and prevalence of naïve T cell subsets. Splenocytes from naïve WT and Anp32b KO mice were isolated and single cell suspensions were cultured for 72 hours without further treatment (upper panel), treated for 72 hours with CD3/CD28 beads (middle panel) or treated for 72 hours with Concanavalin A (ConA; lower panel) and subsequently analyzed with respect to T cell differentiation status, viability and proliferation. Data are mean ± SEM. Anp32b WT (n = 4) and Anp32b KO (n = 4).

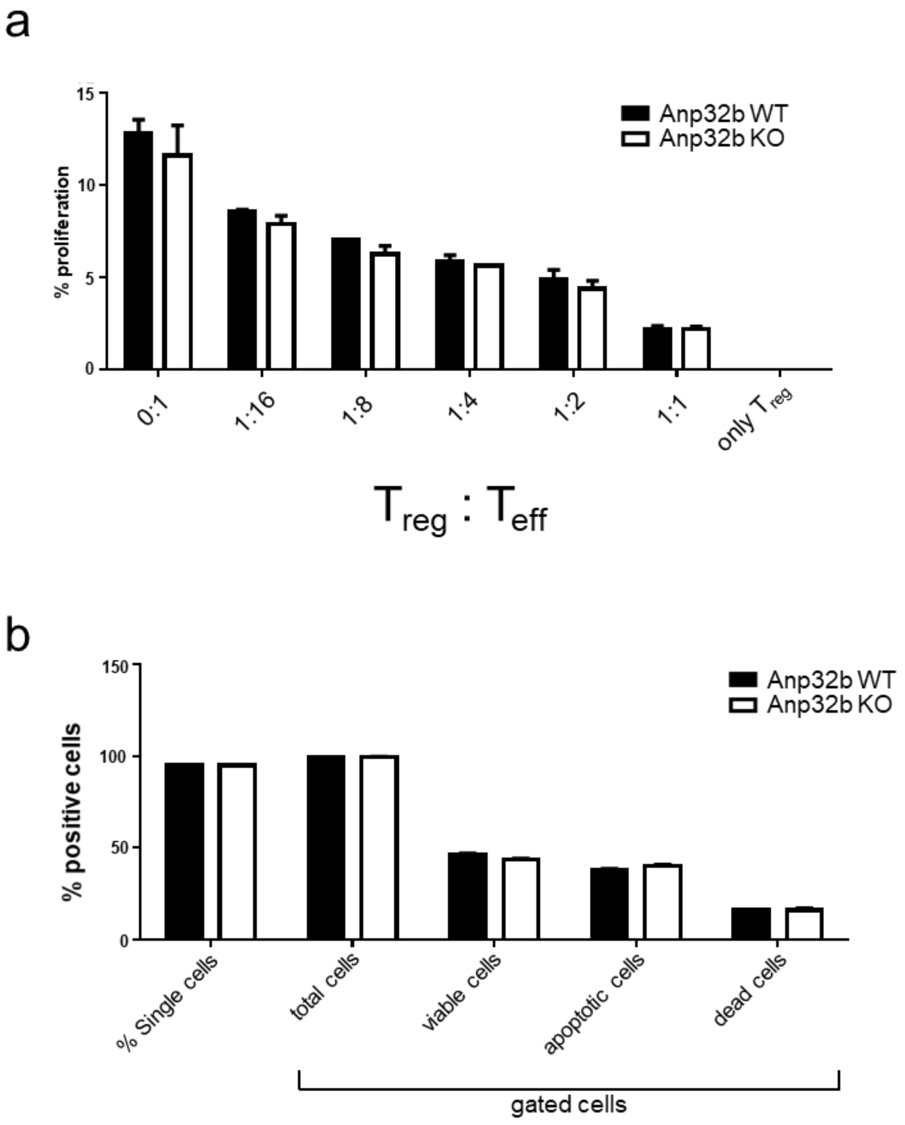

Fig. S7. Suppressor activity of splenic regulatory T cells from wild type (WT) and Anp32b KO mice at day 60 after EAE induction. (a) Cells were isolated and single cell suspensions were subjected to an *in vitro* suppression assay using the indicated T_{reg}:T_{eff} ratios to investigate T_{reg} functionality. **(b)** Viability of splenic cells isolated on day 60 after EAE induction. Data are mean ± SEM. Anp32b WT (n = 2) and Anp32b KO (n = 3).

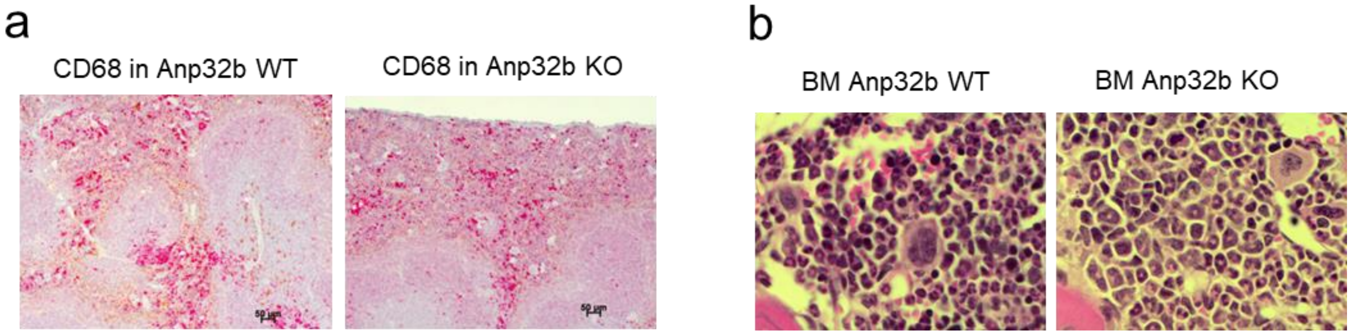

Fig. S8. Immunohistochemistry (a) Immunohistochemical detection of $CD68^{+}$ myelocytes in spleens using a monoclonal antibody specifically staining the CD68 surface molecule on mononuclear cells. **(b)** Haematoxyline-eosine (HE) staining of bone marrow (BM) derived from femurs of Anp32b KO and WT animals.

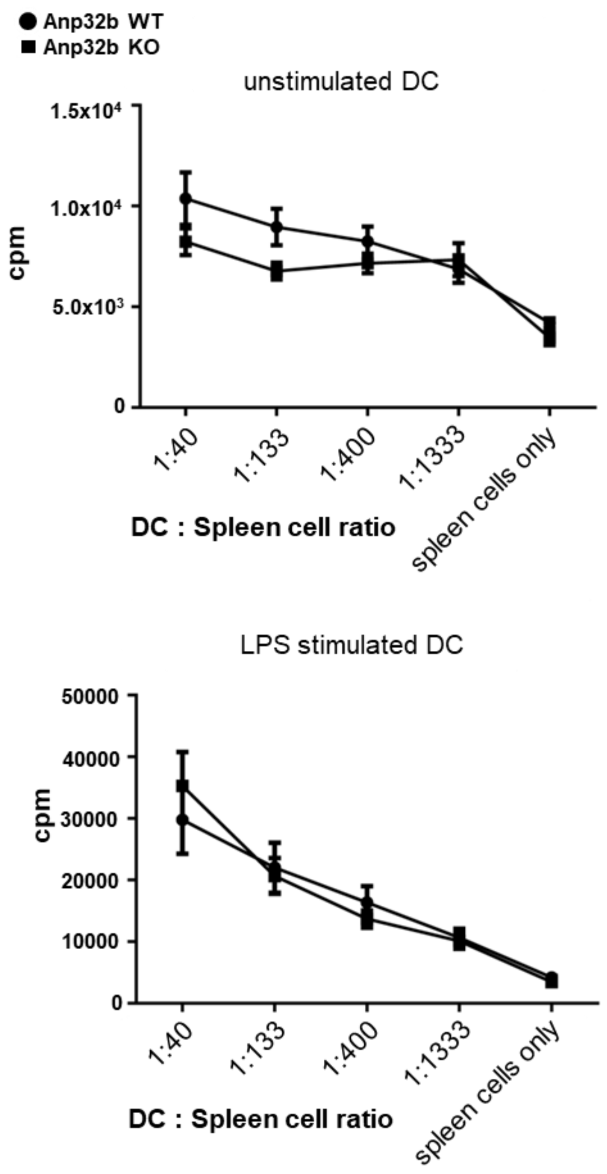

Fig. S9. Allogeneic dendritic cell mediated T cell activation in Anp32b KO mice. A total of 4 x 10^5 splenic cells from Anp32b wild type (WT) or Anp32b KO mice were incubated for 3 days with different numbers of matured DCs generated from BALB/c progenitors in a MLR. DCs were left unstimulated or matured with 100 ng/ml LPS before co-cultivation. Proliferation of allogeneic T cells was analyzed by $^{[3H]}$methyl-thymidine uptake of proliferating cells. Anp32b WT (n = 4) and Anp32b KO (n = 4); pooled data from four independent experiments are shown. Data are mean ± SEM.

Anp32b KO   Anp32b WT

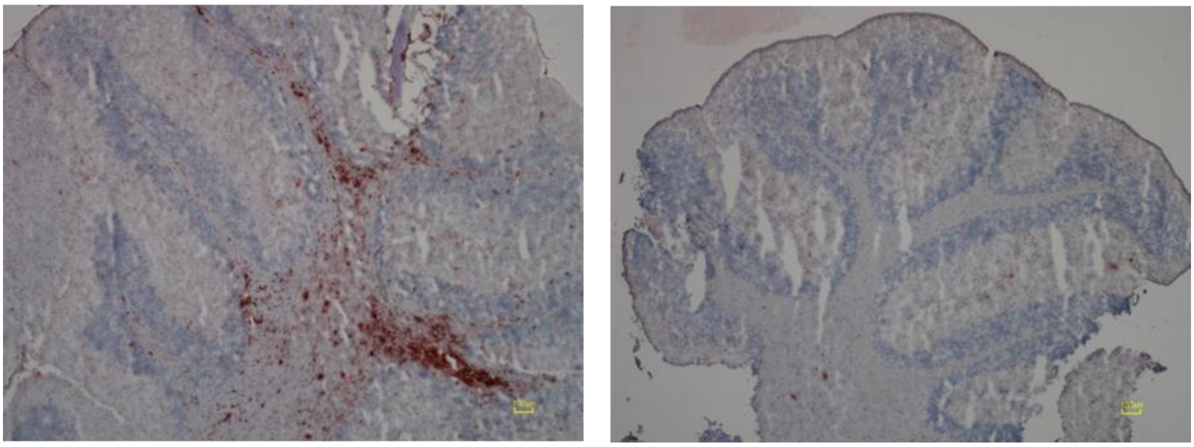

Fig. S10. CD45+ lymphocyte infiltration into brain at day 60 after EAE induction.

Anp32b wild type (WT) or Anp32b KO mice were subjected to EAE induction by injection of MOG peptide. At day 60 of EAE, sections of the brain from Anp32b WT and Anp32b KO mice were stained for CD45+ infiltrates. These experiments were performed at least three times. Data represent a typical experiment.

Supplementary Table 1: Clinical chemistry of serum of WT and ANP32B KO mice

sample ID:	Glucose (mg/dL)	Alkaline Phosphatase ALP (IU/L)	Alanine Aminotransferase ALT (IU/L)	Total Bilirubin TBIL (mg/dL)	Enzymatic Creatinine (mg/dL)	Lactate Dehydrogenase LD (IU/L)
animal #1 WT	174	16	32	0,3	0,12	308
animal #2 WT	171	17	21	0,3	0,06	553
animal #3 KO	175	30	25	0,3	0,07	323
animal #4 WT	131	18	16	0,4	0,09	241
animal #5 KO	156	18	19	0,4	0,06	177
animal #6 KO	167	21	32	0,3	0,07	231
animal #7 WT	164	36	22	0,3	0,08	273

Table S1. Clinical chemistry of Anp32b KO mice serum. Serum of wild type (WT) and Anp32b KO mice was taken and analyzed for serologic parameters as indicated.
